# Experimental evaluation of the accuracy of skin dose calculation for a commercial treatment planning system

**DOI:** 10.1120/jacmp.v9i1.2792

**Published:** 2008-01-28

**Authors:** Laurence E. Court, Roy B. Tishler, Aaron M. Allen, Hong Xiang, Mike Makrigiorgos, Lee Chin

**Affiliations:** ^1^ Department of Radiation Oncology Dana–Farber/Brigham & Women's Cancer Center Boston Massachusetts U.S.A.

**Keywords:** Skin dose, dose calculations, MOSFET

## Abstract

The present work uses the Eclipse treatment planning system (TPS) to investigate the accuracy of skin dose calculations. Micro‐MOSFETs (metal oxide semiconductor field effect transistors) were used to measure skin dose for a range of irradiation conditions (open fields, physical wedges, dynamic wedges, various source‐to‐surface distances) for 6‐MV and 10‐MV beams, and the results were compared with the calculated mean dose to a “skin” structure 2 mm thick for semi‐cylindrical phantoms (representative of a neck or breast). Agreement between the calculated and measured skin dose values was better than ±20% for 95% of all measured points (6‐MV and 10‐MV X‐ray spectra alike). For a fixed geometry, the TPS correctly calculated relative changes in dose, showing that minimization of skin dose in intensity‐modulated radiation therapy will be effective in Eclipse.

PACS numbers: 87.53.Bn, 87.53.Dq, 87.66.Pm, 87.66.Xa

## I. INTRODUCTION

Skin dose can be the limiting factor in radiation therapy treatments, and it is a fairly common cause of interruptions in radiation therapy treatment. Skin dose is of particular concern when intensity‐modulated radiation therapy (IMRT) is used to treat head‐and‐neck cancer.^(1)^ The use of thermoplastic head‐and‐neck immobilization devices increases skin dose,^(^ ^1^ ^,^ ^2^ ^)^ and the use of multiple tangential beams (common in IMRT treatments) can also increase it. The chosen planning and optimization strategy can also affect skin dose, with expansion of the planning target volume to include the skin increasing skin dose, and defining the skin as a sensitive structure reducing skin dose.^(^ ^1^ ^,^ ^3^ ^)^

Despite the clinical importance of skin dose, the literature contains little detail concerning the expected accuracy of skin dose calculations. Fraas et al.^(4)^ (American Association of Physicists in Medicine Task Group 53) reported the collective expectations of the Radiation Therapy Committee Task Group members for pass–fail criteria in the buildup region during the commissioning of a treatment planning system (TPS) as 20% of the central ray normalization dose for regular open fields, increasing to 40% for source‐to‐surface distance (SSD) variations, and 50% for wedged fields. Using radiochromic film, Chung et al.^(5)^ reported that two TPSs [${\text{Pinnacle}}^{3}$ (Philips Medical Systems, Andover, MA) and CORVUS (North American Scientific, Chatsworth, CA)] overestimated surface dose by 7.4%−18.5%. Using thermoluminescent dosimeters and parallel‐plate ionization chambers, Mutic and Low^(6)^ found that a tomotherapy TPS underestimated doses to the surface and first few millimeters below the surface by approximately 15%.

The objective of the present work was to evaluate the accuracy of skin dose calculations in the Eclipse TPS (Varian Medical Systems., Palo Alto, CA) for a wide range of irradiation conditions. In particular, we looked to answer two questions:

- First, by comparing experiments and calculations for a range of irradiation conditions typical in radiation therapy, we wanted to be able to quote the accuracy of skin dose calculations. Physicians often ask planners this important clinical question, particularly for head‐and‐neck treatments, and it is one that has not been evaluated in the literature.
- Second, we wanted to try to predict how the calculation uncertainties would affect the minimization of skin dose during IMRT optimization. For optimization to be reliable, calculated and measured skin dose must be correlated, even if the calculation contains a systematic error.

The literature has yet to address these important clinical questions for the Eclipse TPS. That system is very popular, with approximately 6000 stations installed at 2000 sites worldwide (personal communication, Varian Medical Systems, November 2007).

To answer our questions, we used MOSFET (metal oxide semiconductor field effect transistor) dosimeters (micro‐MOSFET, model TN‐502RDM: Thomson–Nielson Electronics, Nepean, ON) to measure skin dose for 6‐MV and 10‐MV photon spectra, a range of jaw‐defined field sizes, physical and dynamic wedges, IMRT fields, a range of SSDs, and a range of incident angles. We then compared this experimental data with Eclipse calculations. The resulting knowledge will be useful when evaluating and comparing patient plans and also when developing optimization strategies for IMRT (for example, whether the use of a hard constraint for skin dose is sensible).

## II. MATERIALS AND METHODS

### A. Choice of radiation detector

Skin dose measurements can have significant uncertainties. For example, Quach et al.^(7)^ reported that measurements of surface dose by MOSFET, thermoluminescent dosimeter, and film can disagree by up to 50%−60%. In practice, surface (or skin) dose is often taken as the dose measured by whichever dosimeter is used.^(8)^

We recently addressed the question of the accuracy of skin dose measurements made using MOSFETs.^(9)^ To do so, we used carefully commissioned Monte Carlo techniques to calculate the mean dose to a surface volume 2‐mm thick in the region of interest, and we compared those results with experimental dose measurements made by MOSFET. We found that, by embedding the MOSFET in 1 mm of bolus material, the angular dependence effect of the MOSFETs could be reduced, giving readings with a total error of approximately 6%. That error is small as compared with the expectations of Fraas et al.^(4)^ regarding the accuracy of the TPS skin dose calculations (up to 50%). Therefore, MOSFETs are an appropriate choice when measuring skin dose for a range of irradiation conditions.

The micro‐MOSFETs in the present study were used in standard bias setting, for a normal sensitivity of approximately 1 mV/cGy. (References 10 – 13 provide a full description of MOSFETs.) The overall physical size of the sensors is 1.0×1.0×3.5 mm, and the actual sensitive volume is 0.2 mm×0.2 mm×0.5 μm. Signals were read using a wireless mobile MOSFET reader (Model TN‐RD‐16: Thomson–Nielson Electronics), controlled by remote dose‐verification software running on a personal computer. All dose measurements were carried out with the flat side of the MOSFET placed to face the beam. A full description of MOSFETs can be found in references 10–13. The MOSFETs were calibrated before each experimental session by first placing the MOSFET at the depth of maximum dose (1.5 cm and 2.5 cm for 6‐MV and 10‐MV beams respectively) in a Solid Water (Gammex rmi, Middleton, WI) and bolus phantom (bolus sheets were placed on both sides of the MOSFET to minimize air gaps). The MOSFETs were then irradiated with 100 MU in a 10×10‐cm field, and calibration factors in cGy/mV were obtained.

### B. Phantom

**Figure 1:**
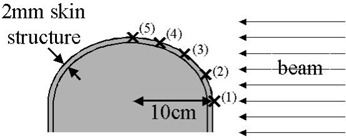
The curved phantom used for the experiments reported here, showing the irradiation geometry and measurement points.

The semi‐cylindrical (12 cm diameter) Solid Water phantom used for this study (Fig. 1) is representative of a neck or breast. The first centimeter of the phantom surface was bolus material. Marks and radiopaque fiducials were used to identify measurement positions on the surface of the phantom. The phantom then underwent computed tomography (CT) scanning, yielding CT pixels 1.3 mm×1.3 mm×2.5 mm, which is representative of the imaging parameters used in our clinic.

### C. Irradiation of the phantom

Table 1 shows the various parameters investigated. The investigations were conducted using a Varian 21Ex at the Dana–Farber Cancer Institute. Calibrated MOSFETs were placed at the measurement points (Fig. 1), embedded in the first millimeter of the surface bolus material. The phantom was irradiated at a gantry angle of 90 degrees, using combinations of the study parameters. Because a curved phantom was used, the effect of irradiation angle is inherently included. For each parameter, the SSD at the central axis was set to 80, 90, and 100 cm, simulating the variations that can be expected in clinical practice. The exact SSD depended on the measurement point, and it varied from 76 cm to 110 cm. All experiments, with the exception of the IMRT fields, were repeated for 6‐MV and 10‐MV X‐ray spectra. The IMRT fields were 6‐MV fields, with the central axis set to 90 cm. The IMRT fields originated in a previous study^(3)^ that investigated planning techniques to minimize skin dose in head‐and‐neck IMRT. The difference between the various plans was the extent to which calculated dose to a skin structure was minimized.

**Table 1:** The various parameters used for dose calculations and measurements

Parameter	Values
Jaw‐defined field size (cm)	3×3, 5×5, 10×10, 15×15, 17×15, 20×20
Physical wedge (degrees)	15, 30, 45, 60
Dynamic wedge (degrees)	15, 30, 45, 60
Dynamic IMRT (6 MV only)	Cumulative dose from multiple beams

IMRT=intensity‐modulated radiation therapy.

### D. TPS calculations

According to reports from the International Commission on Radiological Protection^(14)^ and the International Commission on Radiation Units and Measurements,^(15)^ the recommended depth for practical dose assessments is 0.07 mm. That depth corresponds approximately to the interface between the epidermis and dermis layers of the skin^(^ ^8^ ^,^ ^16^ ^)^ (0.05 – 1.5 mm, depending on the anatomic location). That depth is very difficult to measure or to calculate using most TPSs. For practical reasons in the present work, we therefore chose to define the skin dose as the mean dose to the surface volume (2 mm thickness) in the region of interest.

Separate plans were created in Eclipse for each experimental setup. Skin doses were calculated by first creating a surface structure 2 mm×10 mm×10 mm centered on the measurement mark (as seen using fiducials in the CT images). Skin dose was calculated as the mean dose to this defined structure. The dose calculation grid was set to 2.5 mm, because that size is the one used for most clinical cases.

All calculations were performed using the Eclipse pencil‐beam calculation algorithm. That algorithm first calculates dose at the dose grid points, forcing the dose at points outside the body structure to zero and then interpolating between grid points.

The measured and calculated doses were compared, and the differences were expressed as percentages of the measured dose. Agreement between the Eclipse calculations and the MOSFET measurements was then investigated further for dynamic IMRT fields for a single central axis SSD (90 cm), looking at a single point. The purpose was to investigate the uncertainties in calculated skin dose that may be experienced when IMRT plans are optimized for a single patient (that is, fixed geometry).

## III. RESULTS

Table 2 compares the mean, standard deviation, and range of differences for the various irradiation parameters. Overall, agreement was better than 10% for 75% of all measurement points at 6 MV and 63% of points at 10 MV. Agreement was better than 20% for 94% of the measurement points at 6 MV and 96% of the points at 10 MV.

**Table 2:** Results of the differences between Eclipse skin dose calculations and MOSFET (metal oxide semiconductor field effect transistor) measurements

	Difference^a^ (%)		
	Mean / median	SD	Range
6 MV			
Open field	−4.0/−4.5	13	−26 to +17
Physical wedge	−3.3/−4.5	10	−24 to +14
Dynamic wedge	−7.2/−8.7	10	−27 to +7
IMRT (all SSDs)	4.0/−3.0	9	−15 to +20
Overall	−4.1/−5.9	11	−27 to +20
Single point IMRT	−15.2/−11.4	5	−20 to −7
10 MV			
Open field	−2.0/−2.8	15	−26 to +26
Physical wedge	−6.2/−8.1	9	−21 to +13
Dynamic wedge	−4.2/−2.7	8	−18 to +11
Overall	−3.9/−5.1	11	−26 to +26

a Negative numbers indicate that measured values were lower than those calculated by the treatment planning system. Percentages are given relative to the measured dose.

SD=standard deviation; IMRT=intensity‐modulated radiation therapy; SSD=source to surface distance.

**Figure 2:**
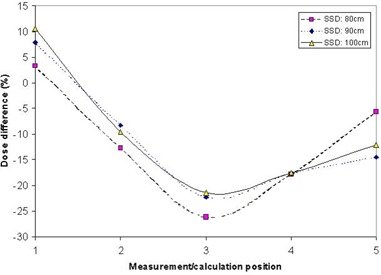
Comparison of the calculated and measured doses for multiple points (open fields) for a 6‐MV beam at various isocenter source‐to‐surface distances (SSDs). The position refers to Fig. 1.

Fig. 2 shows the dose difference (Eclipse vs. MOSFET) for three different setup positions (different SSDs) for various MOSFET positions (shown in Fig. 1) for the 6‐MV beam. The measurement and calculation position can be seen apparently to have a larger effect on the agreement than the SSD does.

**Figure 3:**
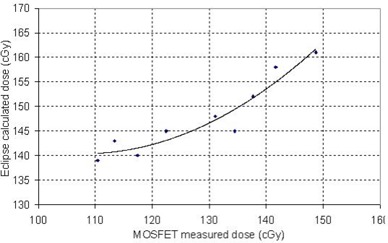
Comparison of the calculated and measured dose for a single point for cumulative intensity‐modulated radiation therapy fields. The curve is a second‐order polynomial fit to the data. Note that the ranges of the two axes are the same, but that an offset occurs between the calculated and the measured data. MOSFET=metal oxide semiconductor field effect transistor.

Table 2 also shows the results for a single point, with fixed SSD, in 10 IMRT plans. Fig. 3 shows a graph of these results. It can be seen from the graph that, although a systematic difference of approximately 13% exists between the Eclipse calculations and the experimental points, Eclipse models the changes in skin dose for the various plans fairly well. That is, a real increase in dose (that is, measured dose) produces an increase in calculated dose. The difference between the Eclipse calculations and a second‐order polynomial fit to the data was −0.3%±1.6% (one standard deviation), with a range of −2%±3%.

## IV. DISCUSSION

For most clinical cases, the data indicate that the Eclipse skin dose calculation is accurate within ±20% (95% of all measurement points). In situations in which more accuracy in the data is needed, on‐patient dose measurements are necessary. Application of these data to other centers will depend on differences in how the dose data for their linear accelerator are obtained and how the TPS is commissioned. In our case, the TPS was commissioned using data supplied by the vendor (Varian's “golden beam” data). Those data were compared with dose distributions [percentage depth doses (PDDs), profiles, isodose curves] measured using a moving ion chamber in a three‐dimensional water phantom (Scanditronix–Wellhofer, Nuremburg, Germany). We also used a parallel‐plate chamber to measure doses near the surface. Table 3 gives the differences between the PDDs in the TPS and those in the experimental parallel‐plate measurements.

**Table 3:** Differences between percentage depth dose (PDD) measured using a parallel‐plate chamber and PDDs used in the treatment planning system

	Difference^a^ (%)	
Depth (mm)	6‐MV Beam	10‐MV Beam
0	−25	−17
2	9	7
4	14	12

a Relative to the maximum dose. Negative numbers indicate that measured values were lower than those calculated by the treatment planning system.

Knowing the accuracy of the skin dose calculation in Eclipse allows for a sensible comparison of skin dose between patients or plans. Importantly, Eclipse seems to accurately model changes in skin dose between IMRT plans, meaning that it can be expected to correctly minimize the skin dose when the skin is defined as a critical structure in those plans. Systematic differences—which depend on measurement point, SSD, and so on—mean that the use of a hard absolute constraint for skin dose (for example, keeping skin dose strictly below 60% of prescription) should be approached with caution. Instead, it may be more advisable to aim for a dose range (for example, 50% – 70% of prescription) or to minimize the skin dose as much as possible (without a specific goal dose), while maintaining specific doses to deeper targets (which are more accurately modeled).

The steep dose gradient at the surface can make reliable dose calculations difficult. Depending on the CT and calculation grid parameters, the interplay between pixel size, pixel location, exact phantom (or patient) location, contour grid, and dose calculation grid can be expected to have an important effect on the calculated doses. This interplay is probably one of the main causes for the importance of position rather than SSD in the dose calculation accuracy (Fig. 2). Depending on position, the interplay will be different, resulting in different calculations of skin dose. This finding may be particularly true in the case of flat phantoms, in which no opportunity for averaging of the effects occurs. Nevertheless, for the parameters tested here, we found reasonable agreement between the experimental and calculated skin doses. Calculations for parameters outside of those tested here should still be treated with caution.

## V. CONCLUSION

Agreement between skin doses calculated by Eclipse and those measured by MOSFET in hemispheric phantoms was within ±20% for 95% of all measurement points. Eclipse models relative increases and decreases in skin dose from different IMRT plans reasonably well, and so IMRT optimization can be expected to be successful in reducing skin dose. However, an absolute goal for skin dose should be considered with caution.

## Supporting information

Supplementary Material
