# Supplementary material for: Experimental evaluation of the accuracy of skin dose calculation for a commercial treatment planning system
Source: J Appl Clin Med Phys. 2008 Jan 28;9(1):29–35. doi: 10.1120/jacmp.v9i1.2792 (PMC5721536; doi:10.1120/jacmp.v9i1.2792)
Supplement: Supplementary file 1 — Supplementary Material [file ACM2-9-29-s001.doc]

**Experimental evaluation of the accuracy of skin dose calculation for a commercial treatment planning system**

**Laurence E Court, Roy B Tishler, Aaron M Allen,**

**Hong Xiang, Mike Makrigiorgos, Lee Chin**

## Department of Radiation Oncology

*Dana-Farber / Brigham & Women’s Cancer Center*

*44 Binney Street, Boston MA 02115 USA*

[lcourt@lcroc.harvard.edu](mailto:lcourt@lcroc.harvard.edu)

Running title: Accuracy of skin dose calculations

Key words: skin dose, dose calculations, MOSFETs

PACS numbers: 87.53.Bn, 87.53.Dq, 87.66.Pm, 87.66.Xa

**Abstract:** This work investigates the accuracy of skin dose calculations using the Eclipse treatment planning system. Skin dose was measured using micro-MOSFETs for a range of irradiation conditions (open fields, physical wedges, dynamic wedges, different SSDs) for 6MV and 10MV beams, and compared with the mean dose calculated to a 2mm thick “skin” structure for semi-cylindrical phantoms (representative of a neck or breast). Agreement between the calculated and measured skin dose values was better than ±20% for 95% of all measured points (both 6MV and 10MV x-ray spectra). For a fixed geometry, the TPS correctly calculated relative changes in dose, showing that minimization of skin dose in IMRT will be effective in Eclipse.

Key words: skin dose, dose calculations, MOSFETs

PACS numbers: 87.53.Bn, 87.53.Dq, 87.66.Pm, 87.66.Xa

# I. INTRODUCTION

Skin dose can be the limiting factor in radiation therapy treatments, and is a fairly common cause for interruptions in radiation therapy treatment. Skin dose is of particular concern when IMRT is used to treat head-and-neck cancer^(1)^. It is increased by the use of thermoplastic head and neck immobilization devices^(1,2)^, and can also be increased by the use of multiple tangential beams (common in IMRT treatments). The planning and optimization strategy used can also impact skin dose, where expanding the PTV to include the skin will increase skin dose, and using the skin as a sensitive structure in the optimization can reduce skin dose^(1,3)^.

In spite of the clinical importance of skin dose, there is little literature available detailing the expected accuracy of skin dose calculations. Fraas et al^(4)^(AAPM Task Group 53) reported the collective expectations of the task group members for pass-fail criteria in the build-up region when commissioning a TPS as 20% of the central ray normalization dose for regular open fields, increasing to 40% for SSD variations and 50% for wedged fields. Chung et al^(5)^, using radiochromic film, reported that two TPS (PINNACLE3 and CORVUS) overestimated surface dose by 7.4% to 18.5%. Mutic and Low^(6)^, using TLD and parallel-plate ionization chambers, found that the tomotherapy TPS underestimated doses to the surface and first few millimeters below the surface by around 15%.

The objective of this work is to evaluate the accuracy of skin dose calculations using the Eclipse TPS (Varian Medical Systems Inc., Paol Alto, CA) for a wide range of irradiation conditions. In particular, we look to answer two questions:

- Firstly, by comparing experiment and calculations for a range of irradiation conditions typical in radiation therapy, we would like to be able to quote the accuracy of skin dose calculations. This is an important clinical question that planners are often asked by physicians, particularly for head and neck treatments, and is one that has not been evaluated in the literature.
- Secondly, we tried to predict how the calculation uncertainties will impact the minimization of skin dose during IMRT optimization. For optimization to be reliable, calculated and measured skin dose must be correlated, even if there is a systematic error in the calculation.

These are important clinical questions that have yet to be addressed in the literature for the Eclipse treatment planning system. This is a very popular system, with approximately 6000 Eclipse stations installed at 2000 sites worldwide (source: email from Varian Medical Systems, November 2007). To answer these questions, we used MOSFETs (micro-MOSFET dosimeters model TN-502RDM, Thomson-Nielson Electronics Ltd., Nepean, Canada) to measure the skin dose for 6MV and 10MV photon spectra, a range of jaw-defined field sizes, physical and dynamic wedges, IMRT fields, a range of SSDs, and a range of incident angles. This experimental data was then compared with Eclipse calculations. This knowledge will be useful when evaluating and comparing patient plans, and also will be useful when developing optimization strategies for IMRT (e.g. whether or not it is sensible to use a hard constraint for skin dose).

II. MATERIALS AND METHODS

### Choice of radiation detector

Skin dose measurements can have significant uncertainties. For example, Quach et al^(7)^ reported that surface dose measured using MOSFET, TLD and film can disagree by up to 50-60%. In practice, surface (or skin) dose is often taken as the dose measured by whichever dosimeter is used^(8)^. We^(9)^ recently addressed the question of how accurate skin dose measurements are when made using MOSFETs. We did this by using carefully commissioned Monte Carlo techniques to calculate the mean dose to the surface 2mm thick volume in the region of interest, and compared this with experimental dose measurements using MOSFETs. It was found that by embedding the MOSFET in 1mm of bolus material it is possible to reduce the effect of the angular dependence of the MOSFETs, giving a total error in the readings of ~6%. This is small compared with the expectations of Fraas et al^(4)^ regarding the accuracy of the TPS skin dose calculations of up to 50%. MOSFETs are, therefore, an appropriate choice when measuring skin dose for a range of irradiation conditions.

The MOSFETs used in this study (micro-MOSFETs, Thomson-Nielson Electronics Ltd, Canada) were used in standard bias setting, giving a normal sensitivity of ~1mV/cGy. The overall physical size of the sensors is 1.0 x 1.0 x 3.5 mm^3^, and the actual sensitive volume is 0.2 mm x 0.2mm x 0.5μm. Signals were read out using a wireless mobile MOSFET reader (Model TN-RD-16, Thomson-Nielson), controlled with remote dose verification software running on a PC. All dose measurements were carried out with the flat side of the MOSFET placed to face the beam. A full description of MOSFETs can be found in references 10-13. The MOSFETs were calibrated before each experimental session by first placing the MOSFET at dmax (1.5 or 2.5cm for 6MV and 10MV beams, respectively) in a solid water/bolus phantom (bolus sheets were placed on both sides of the MOSFET to minimize air gaps). The MOSFETs were then irradiated with 100MU using a 10 x 10 cm^2^ field, and calibration factors in cGy/mV were obtained.

### Phantom

A semi-cylindrical solid water phantom was used for this study (12cm diameter), as illustrated in figure 1, representative of a neck or breast. The surface 1cm of this phantom was bolus material. Measurement positions were identified on the surface of the phantom using marks and radiopaque fiducials. The phantom was CT scanned, giving CT pixels 1.3mm x 1.3mm x 2.5mm. This is representative of the imaging parameters used in our clinic.

### Irradiation of the phantom

The different parameters investigated are shown in table 1. These were investigated using a Varian 21Ex at Dana-Farber Cancer Institute. Calibrated MOSFETs were placed at the measurement points (seen in figure 1), embedded in the surface 1mm of bolus material. The phantom was irradiated using a gantry angle of 90°, using combinations of these parameters. Because this experiment uses a curved phantom, it inherently includes the impact of irradiation angle. For each parameter, the SSD at central-axis was set to 80, 90 and 100cm, to simulate the variations that can be expected in clinical practice. The exact SSD depended on the measurement point, and varied from 76cm to 110cm. All experiments, with the exception of the IMRT fields, were repeated for 6MV and 10MV x-ray spectra. The IMRT fields were 6MV, with central-axis set to 90cm. The IMRT fields were from a previous study^(3)^ that investigated planning techniques to minimize skin dose in head and neck IMRT. The difference between the different plans was the extent to which calculated dose to a skin structure was minimized.

### Treatment planning system calculations

According to ICRP^(14)^ and ICRU^(15)^ reports, the recommended depth for practical dose assessments is 0.07mm. This corresponds approximately to the interface between the epidermis and dermis layers of the skin^(8,16)^ (0.05 – 1.5mm, depending on the anatomic location). This is very difficult to measure, or calculate using most treatment planning systems. For practical reasons, for this work, we therefore chose to define the skin dose as the mean dose to the surface 2mm thick volume in the region of interest.

Separate plans were created in Eclipse for each experimental setup. Skin doses were calculated by first creating a 2mm thick 10mm x 10mm surface structure centered on the measurement mark (as seen using fiducials in the CT images). For the propose of this study, skin dose was calculated as the mean dose to this structure. The dose calculation grid was set to 2.5mm, as this is the size used for most clinical cases. All calculations used Eclipse’s pencil-beam calculation algorithm. This algorithm first calculates dose at the dose grid points, forcing the dose at points outside the body structure to zero, and then interpolates between grid points. The measured and calculated doses were compared, and the differences were expressed as a percentage of the measured dose.

Agreement between Eclipse calculation and MOSFET measurement was then investigated further for dynamic IMRT fields for a single central-axis SSD (90cm), looking at a single point. The purpose of this study was to investigate the uncertainties in calculated skin dose that may be experienced when IMRT plans are optimized for a single patient (i.e. fixed geometry).

III. RESULTS

Table 2 compares the mean, standard deviation, and range of differences for different irradiation parameters. Overall, agreement was better than 10% for 75% and 63% of all measurement points for 6MV and 10MV spectra, respectively. Agreement was better than 20% for 94% and 96% of the measurement points for 6MV and 10MV spectra, respectively.

Figure 2 shows the dose difference (Eclispe vs. mosfet) for different three different setup positions (different SSDs) for different mosfet positions (positions are shown in figure 1) for the 6MV beam. It can be seen that the position of the measurement/calculation appears to have a larger impact on the agreement than the SSD.

The results for a single point, with fixed SSD, for 10 IMRT plans are also shown in table 2. Figure 3 shows a graph of these results. It can be seen from the graph that although there is a systematic difference between Eclipse and the experimental points of around 13%, Eclipse does model the changes in skin dose from different plans fairly well. That is, a real increase in dose (i.e. measured dose) will result in an increase in calculated dose. The difference between the Eclipse calculations and a second order polynomial fit to the data was –0.3% ±1.6% (1 s.d.), with a range of –2% - +3%.

IV. DISCUSSION

For most clinical cases, this data indicates that the Eclipse skin dose calculation is accurate within ±20% (95% of all measurement points). In situations where more accurate data is needed, on-patient dose measurements are necessary. Application of this data to other centers will depend on differences in how the LINAC dose data is obtained, and how the treatment planning system is commissioned. In our case, the TPS was setup using data supplied by the vendor (Varian – so called “Golden beam” data). This was compared with dose distributions (PDDs, profiles, isodose curves) measured using a moving ion chamber in a 3D water phantom (Wellhofer). Doses near the surface were also measured using a parallel-plate chamber. The differences between the PDDs in the TPS and experimental parallel-plate PDDs are given in table 3.

Knowing the accuracy of the skin dose calculation in Eclipse will allow sensible comparison of skin dose between different patients, or different plans. Importantly, Eclipse seems to accurately model changes in skin dose between different IMRT plans, meaning that it can be expected to correctly minimize the skin dose when the skin is used as a critical structure in IMRT plans. Systematic differences, which depend on measurement point, SSD etc, mean that the use of a hard absolute constraint for skin dose (e.g. strictly keeping skin dose below 60% of the prescription) should be approached with caution. Instead, it might be more advisable to aim for a dose range (e.g. 50-70% of prescription), or to minimize skin as much as possible (without a specific goal dose) while maintaining specific dose to deeper targets (which is more accurately modeled).

The steep dose gradient at the surface can make reliable dose calculations difficult. It can, be expected that, depending on the CT and calculation grid parameters, the interplay between the pixel size, pixel location, exact phantom (patient) location, contour grid, and dose calculation grid will have an important impact on the calculated doses. This is probably one of the main causes for the importance of position (rather than SSD) on the dose calculation accuracy (figure 2) – depending on the position, these interplays will be different, resulting in different calculated skin dose. This might be particularly true for flat phantoms where might be no opportunity for averaging of the effects. In spite of this, for the parameters tested here, there was reasonable agreement between experimental and calculated skin doses. Calculation for parameters outside those tested here should, therefore, be treated with caution.

V. CONCLUSION

Agreement between skin dose calculated by Eclipse and MOSFET measurements was within ±20% for 95% of all measurement points for hemi-spherical phantoms. Eclipse does model relative increases/decreases in skin dose due to different IMRT plans reasonably well, so IMRT optimization can be expected to be successful in reducing skin dose. However, the use of an absolute goal for skin dose should be considered with caution.

REFERENCES

1. Lee N, Chuang C, Quivey JM, et al. Skin toxicity due to intensity-modulated radiotherapy for head-and-neck carcinoma. Int. J. Rad. Biol. Phys. 2002; 53(3): 630-637.

2. Hadley SW, Kelly, R, Lam K. Effects of immobilization mask material on surface dose. J. App. Clin. Med. Phys. 2005; 6(1): 1-7.

3. Court LE, Tisher RB. **Planning Strategies to Reduce Unnecessary Skin Dose in Head and Neck IMRT, Including Experimental Verification (accepted by** Int. J. Rad. Biol. Phys.)

4. Fraas B, Doppke K, Hunt M et al. American Association of Physicists in Medicine Radiation Therapy Committee Task Group 53: Quality assurance for clinical radiotherapy treatment planning. Med. Phys. 1998; 25(10): 1773-1829.

**5. Chung H, Jin H, Dempsey JF et al. Evaluation of surface and build-up region dose for intensity-modulated radiation therapy in head and neck cancer. Med. Phys. 2005; 32(8): 2682-2689.**

6. Mutic S, Low DA, Superficial doses from serial tomotherapy delivery. Med. Phys. 2000; 27(1): 163-163.

7. Quach, J Morales, M J Butson, A B Rosenfeld, P E Metcalfe, Measurement of radiotherapy x-ray skin dose on a chest wall phantom, Med. Phys. 2000; 27(7): 1676-1680.

8. Devic S, Seuntjens J, Abdel-Rahman W et al. Accurate skin dose measurements using radiochromic film in clinical applications. Med. Phys. 2006; 33(4): 1116-1124.

9. Xiang HF, Song J, Chin DWH, Cormack RA, Tishler RB, Makrigiorgos GM, Court LE, Chin LM, Skin dose measurements on phantoms using micro-MOSFET in 6 and 10 MV X-ray beams and comparisons with Monte Carlo calculations. Med. Phys. 2007;34(4): 1266-1273.

10. Chuang CF, Verhey LJ, and Xia P, Investigation of the use of MOSFET for clinical IMRT dosimetric verification. Med. Phys. 2002; 29(6): 1109–1115.

11. Ramaseshan R, Kohli KS, Zhang TJ, et al. Performance characteristics of a micro-MOSFET as an *in vivo* dosimeter in radiation therapy. Phys. Med. Biol. 2004; 49: 4031–4048.

12. Ramani R, Russell S, and O’Brien P, Clinical dosimetry using MOSFETs. Int. J. Radiat. Oncol., Biol., Phys. 1997; 37: 959–964.

13. Soubra M, Cygler J, and Mackay G, Evaluation of a dual bias dual metal oxide-silicon semiconductor field effect transistor detector as radiation dosimeter. Med. Phys. 1994; 21: 567–572.

14 ICRP Publication 60. *Recommendations of the international Commission on Radiological Protection*, Pergamon, Oxford 1991.

15. ICRU, Determination of dose equivalents resulting rom external radiation sources Report No. 39, International Commission on Radiation units and measurement, Washington, DC, 1985.

16. ICRP Publication 23, Anatomical, Physiological and Metabolic Characteristics (Pergamon, Oxford, 1975).

# Figure Legends

Figure 1. The curved phantom used for these experiments, showing the irradiation geometry and measurement points.

Figure 2. Comparison of calculated and measured dose for a multiple points (open fields) for 6MV beam, different isocenter SSDs. The position refers to figure 1.

Figure 3. Comparison of calculated and measured dose for a single point for cumulative IMRT fields. The curve is a second-order polynomial fit to the data. Note that the ranges of the two axis is the same, but there is an offset between the calculated and measured data.

**Tables**

| Parameter | Values |
| --- | --- |
| Jaw-defined field size | 3x3, 5x5, 10x10 15 x 15, (17) x 15, 20x20 |
| Physical wedge | 15°, 30°, 45°, 60° |
| Dynamic wedge | 15°, 30°, 45°, 60° |
| Dynamic IMRT | (cumulative dose from multiple beams, 6MV only) |

Table 1. Different parameters used for dose calculations and measurements

|  | Mean / median  difference (%) | S.D. (%) | Range (%) |
| --- | --- | --- | --- |
| Open field, 6MV | -4.0 / -4.5 | 13 | -26 - +17 |
| Physical wedge, 6MV | -3.3 / -4.5 | 10 | -24 - +14 |
| Dynamic wedge, 6MV | -7.2 / -8.7 | 10 | -27 - +7 |
| IMRT, 6MV (all SSD) | 4.0 / -3.0 | 9 | -15 - +20 |
| Overall, 6MV | -4.1 / -5.9 | 11 | -27 - +20 |
|  |  |  |  |
| Single point IMRT, 6MV | -15.2 / -11.4 | 5 | -20 - -7 |
|  |  |  |  |
| Open field, 10MV | -2.0 / -2.8 | 15 | -26 - +26 |
| Physical wedge, 10MV | -6.2 / -8.1 | 9 | -21 - +13 |
| Dynamic wedge, 10MV | -4.2 / -2.7 | 8 | -18 - +11 |
| OVERALL, 10MV | -3.9 / -5.1 | 11 | -26 - +26 |

Table 2. Results of the differences between Eclipse skin dose calculations and MOSFET measurements. Negative values indicate measurements were lower than TPS calculations. Percentages are given relative to the measured dose.

| Depth (mm) | 6MV beam | 10MV beam |
| --- | --- | --- |
| 0 | -25 | -17 |
| 2 | 9 | 7 |
| 4 | 14 | 12 |

Table 3. Percentage differences between PDD measured using a parallel plate chamber and PDDs used in the TPS. Percentages are given relative to maximum dose. Negative values indicate measurements were lower than TPS calculations.
